# NBP35 interacts with DRE2 in the maturation of cytosolic iron‐sulphur proteins in *Arabidopsis thaliana*

**DOI:** 10.1111/tpj.13409

**Published:** 2017-02-03

**Authors:** Emma L. Bastow, Katrine Bych, Jason C. Crack, Nick E. Le Brun, Janneke Balk

**Affiliations:** ^1^ John Innes CentreNorwichNR4 7UHUK; ^2^ University of East AngliaNorwichNR4 7TJUK; ^3^ Department of Plant SciencesUniversity of CambridgeCambridgeCB2 3EAUK; ^4^ Present address: Glycom A/SDK – 2800 Kgs.LyngbyDenmark

**Keywords:** Fe–S cofactor, cytosol, leaf development, aconitase, aldehyde oxidase, DNA methylation, CIAPIN1, yeast‐two‐hybrid, *Arabidopsis thaliana*

## Abstract

Proteins of the cytosolic pathway for iron‐sulphur (FeS) cluster assembly are conserved, except that plants lack a gene for CFD1 (Cytosolic FeS cluster Deficient 1). This poses the question of how NBP35 (Nucleotide‐Binding Protein 35 kDa), the heteromeric partner of CFD1 in metazoa, functions on its own in plants. Firstly, we created viable mutant alleles of *NBP35* in Arabidopsis to overcome embryo lethality of previously reported knockout mutations. RNAi knockdown lines with less than 30% NBP35 protein surprisingly showed no developmental or biochemical differences to wild‐type. Substitution of Cys14 to Ala, which destabilized the N‐terminal Fe_4_S_4_ cluster *in vitro*, caused mild growth defects and a significant decrease in the activity of cytosolic FeS enzymes such as aconitase and aldehyde oxidases. The DNA glycosylase ROS1 was only partially decreased in activity and xanthine dehydrogenase not at all. Plants with strongly depleted NBP35 protein in combination with Cys14 to Ala substitution had distorted leaf development and decreased FeS enzyme activities. To find protein interaction partners of NBP35, a yeast‐two‐hybrid screen was carried out that identified NBP35 and DRE2 (Derepressed for Ribosomal protein S14 Expression). NBP35 is known to form a dimer, and DRE2 acts upstream in the cytosolic FeS protein assembly pathway. The NBP35–DRE2 interaction was not disrupted by Cys14 to Ala substitution. Our results show that NBP35 has a function in the maturation of FeS proteins that is conserved in plants, and is closely allied to the function of DRE2.

## Significance Statement

Knockout mutants of NBP35 (Nucleotide‐Binding Protein 35 kDa) are embryo lethal, which has precluded functional studies. Here we generated a line with a Cys14 to Ala substitution in the N‐terminal iron‐sulfur (FeS) cluster binding motif, which exhibited a decrease in activities of several cytosolic FeS enzymes. In addition, NBP35 was found to interact with DRE2, a protein upstream in the pathway. Thus we demonstrate that NBP35 has a function in the maturation of FeS proteins.

## Introduction

Iron–sulphur (FeS) clusters are versatile metal cofactors involved in electron transfer, catalytic reactions as well as regulation of gene expression (Beinert, 2000; Lill, 2009). The rhombic Fe_2_S_2_ and the cubane Fe_4_S_4_ clusters are the most common forms, liganded to the protein by three to four cysteine residues. In some FeS proteins, histidine or aspartate are used as ligands in addition to cysteines. FeS clusters are synthesized by dedicated assembly proteins in the plastids, mitochondria and cytosol. Plastids contain the SUF pathway, and mitochondria contain the ISC pathway, each involving at least eight proteins (Couturier *et al*., 2013; Balk and Schaedler, 2014). The assembly process starts with the formation of a persulphide on cysteine desulphurase, which is transferred to a scaffold protein. The persulphide (S^0^) is reduced to sulphide (S^2−^) and combined with iron before transfer of the cluster to a target protein. The cytosol and nucleus harbour another set of seven to eight proteins required for FeS cluster assembly, known collectively as the CIA pathway for Cytosolic Iron–sulphur protein Assembly. The assembly process is dependent on the mitochondria, which are thought to provide persulphide, exported in the form of glutathione trisulphide by the ABC transporter Atm1 in yeast (*Saccharomyces cerevisiae*) or ATM3 in plants (Schaedler *et al*., 2014). It is still unknown how the iron is provided and incorporated in FeS clusters.

The proteins of the CIA pathway have been studied mostly in yeast (Netz *et al*., 2014), but are found in virtually all eukaryotes (Tsaousis *et al*., 2014). The first identified were two P loop NTPases, Cfd1 and Nbp35, that form a heterotetrameric complex (Netz *et al*., 2007). However, plants and algae lack a *CFD1* gene, and purified Nucleotide‐Binding Protein 35 kDa (NBP35) forms a homodimer (Bych *et al*., 2008; Kohbushi *et al*., 2009). Arabidopsis *NBP35* could not replace the yeast homologue in complementation assays, but *in vitro* data suggest that Arabidopsis NBP35 has a scaffold function similar to the yeast Npb35/Cfd1 complex. Specifically, the C‐terminus of NBP35 binds a labile FeS cluster that can be transferred to an apoprotein (Netz *et al*., 2007; Bych *et al*., 2008). A second, stable ferredoxin‐type FeS cluster is bound to the N‐terminal domain of NBP35.

The other CIA proteins can be divided into two functional units based on protein interactions. A complex of four proteins, Nar1, Cia1, Cia2 and Met18 (yeast nomenclature), has been placed experimentally downstream of Nbp35/Cfd1. Cia2 and Met18 interact directly with FeS apoproteins in yeast, mammals and plants (Netz *et al*., 2014; Duan *et al*., 2015; Wang *et al*., 2016). Functioning upstream of Nbp35/Cfd1 is a complex of two CIA proteins, the diflavin reductase Tah18 and the FeS‐binding Dre2 (Derepressed for Ribosomal protein S14 Expression, also known as Anamorsin or CIAPIN1). They have a redox function with electrons passing from NADPH via Tah18 to Dre2.

There are only a limited number of functional studies on CIA proteins in plants because all genes except *MET18* are essential. A non‐lethal mutation in the *CIA2/AE7* gene was found in a screen for mutants with altered leaf polarity (Yuan *et al*., 2010). *ae7* mutants also have decreased activities of cytosolic and nuclear FeS enzymes, and increased DNA damage (Luo *et al*., 2012). *met18* mutants do not have a visible growth phenotype, but are deficient in demethylating DNA as a result of decreased activity of the DNA glycosylase ROS1, a Fe_4_S_4_ enzyme, and possibly other demethylation pathways (Duan *et al*., 2015; Wang *et al*., 2016).

Here we present a viable mutant allele of *NBP35* in Arabidopsis, and show that the protein is required for cytosolic aconitase (ACO) and aldehyde oxidase (AldOx) activities, but that DNA glycosylase and xanthine dehydrogenase (XDH) are less affected. The function of NBP35 is necessary during reproductive and vegetative development, and affects leaf polarity. In a yeast‐two‐hybrid screen with an Arabidopsis cDNA library, DRE2 was revealed as a protein interaction partner of NBP35.

## Results

### Less than 30% NBP35 protein is sufficient for vegetative growth and the maturation of cytosolic FeS proteins

**Figure 1:**
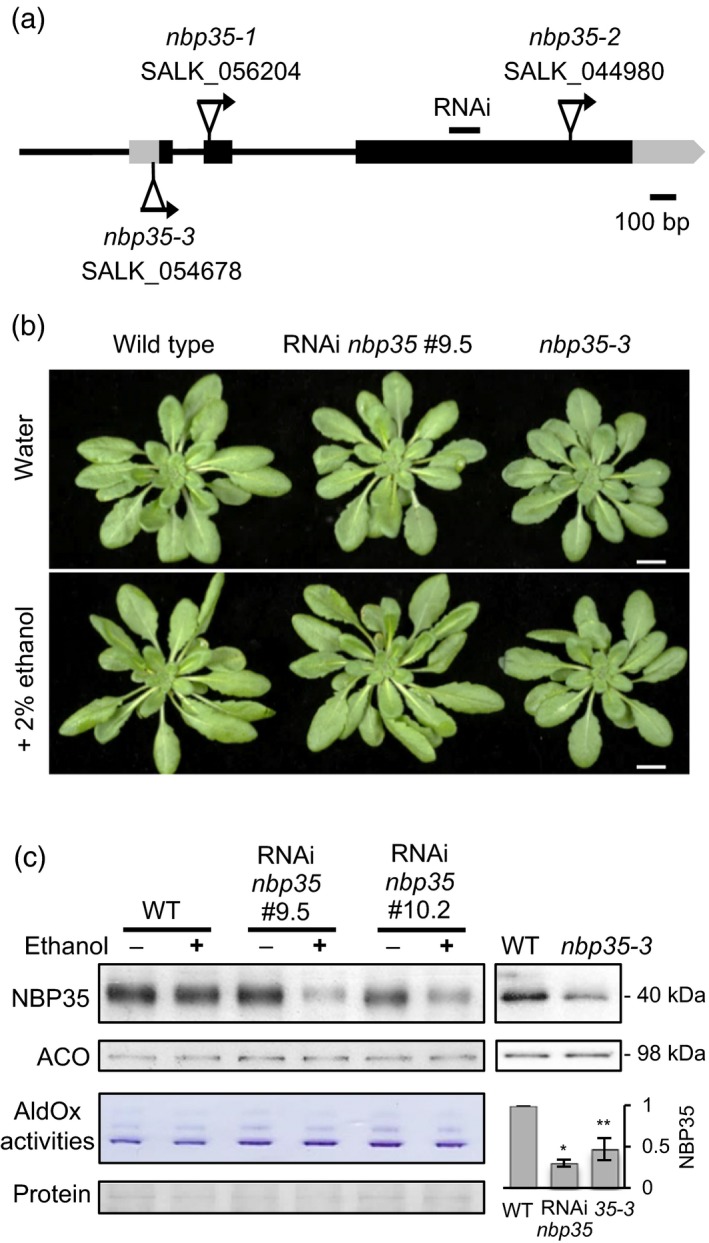
Downregulation of *NBP35* does not affect growth or FeS enzyme activities. (a) Schematic representation of the Arabidopsis *NBP35* gene (*AT5G50960*) and T‐DNA insertions. Arrows indicate the left border of the T‐DNA. The T‐DNA insertion sites were confirmed by sequencing: +1 in exon 2 for *nbp35‐1* and −49 for *nbp35‐3*. (b) Five‐week‐old leaf rosettes of wild‐type, the ethanol‐inducible RNAi *nbp35* line and *nbp35‐3* after treatment with 2% ethanol for 2 weeks, or water as control, as indicated. Plants were grown under short‐day conditions to extend the vegetative growth stage. Scale bar: 1 cm. (c) Protein blot analysis of NBP35 and aconitase (ACO) in wild‐type (WT), two independent lines of RNAi *nbp35* and the T‐DNA insertion mutant *nbp35‐3*. Plants were treated with ethanol as indicated. Aldehyde oxidase (AldOx) activities and Ponceau‐S staining for protein loading are also shown. Bottom right, fraction of NBP35 protein in RNAi line *nbp35* #9.5 (+2% ethanol) and *nbp35‐3* (*35‐3*) normalized to wild‐type. Error bars indicate the standard deviation of three independent experiments. *P < 0.01, **P < 0.001.

Previous studies showed that T‐DNA insertion in the coding sequence of the *NBP35* gene is embryo lethal, for instance in the *nbp35‐1* and *nbp35‐2* mutant alleles (Figure 1a; Bych *et al*., 2008; Kohbushi *et al*., 2009). In contrast, the *nbp35‐3* allele, which has a T‐DNA insertion in the 5′UTR (‐49 nt), is viable (Bych *et al*., 2008; Nakamura *et al*., 2013). Using specific antibodies, we found that NBP35 protein levels in leaves of *nbp35‐3* are 47 ± 13% of wild‐type levels, but the plants have no obvious phenotype under standard growth conditions (Figure 1b and c).

To obtain alleles with a stronger depletion of NBP35, we silenced expression using an ethanol‐inducible RNAi sequence. Of 12 independent lines, two were selected for further study because of a strong decrease in NBP35. Leaf extracts of RNAi plants treated with ethanol for 2 weeks contained 29 ± 5% NBP35 of wild‐type levels as measured by semi‐quantitative protein blot analysis with NBP35‐specific antibodies (Figures 1c and S1). NBP35‐depleted plants looked similar to wild‐type or water‐treated controls (Figure 1b). To investigate if there was any effect on the maturation of cytosolic FeS enzymes, we analysed the levels of ACO by immunolabelling and the activities of AldOx by in‐gel staining (Figure 1c). Neither ACO nor AldOx were decreased in the RNAi lines or in *nbp35‐3* compared with wild‐type leaves.

### Cys14 to Ala substitution in NBP35 decreased the activities of selected FeS enzymes in the cytosol

**Figure 2:**
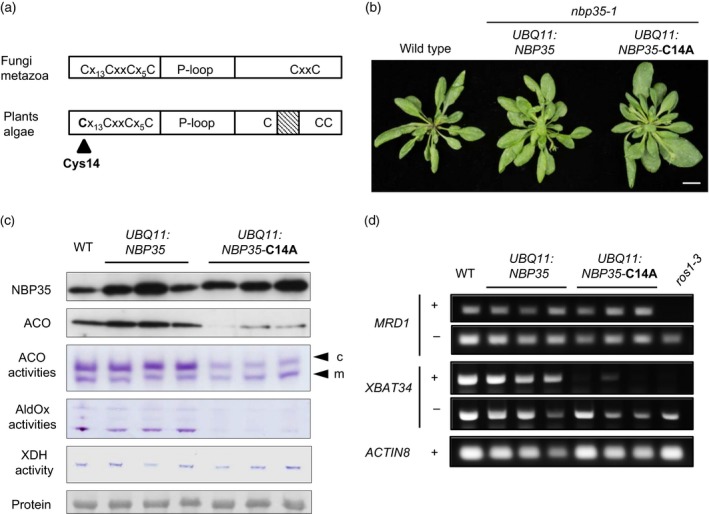
Cys14 to Ala substitution in NBP35 leads to decreased FeS enzyme activities. (a) Schematic of NBP35 protein domain structure including the N‐terminal and C‐terminal cysteine motifs flanking the P‐loop domain. (b) Leaf rosette development in 25‐day‐old plants with either *NBP35* or *NBP35‐*C14A expressed from the *UBQ11* promoter in the absence of endogenous *NBP35* (homozygous for *nbp35‐1*). Plants were grown under long‐day conditions. Scale bar: 1 cm. (c) Protein blot analysis of NBP35 and aconitase (ACO), as well as activity staining for ACO, aldehyde oxidase (AldOx) and xanthine dehydrogenase (XDH) activities in wild‐type (WT) and three independent lines each of *UBQ11:NBP35* and *UBQ11:NBP35‐*C14A in the *nbp35‐1* background. (d) McrBC‐PCR assay on genomic DNA samples from the indicated lines using two selected loci (*MRD1* and *XBAT34*). Lack of a PCR product after digestion with the methylation‐dependent endonuclease McrBC (+) indicates hyper‐methylation due to inactive ROS1. The *ros1‐3* mutant was included for comparison. PCR with *ACTIN8*‐specific primers served to confirm the levels of genomic DNA.

As an alternative approach to create a viable mutant allele of *NBP35*, we decided to replace the endogenous NBP35 protein with a mutant version. We targeted amino acid residues that are evolutionary conserved but not essential in yeast Nbp35 (Netz *et al*., 2012). Cys27 is a ligand of the N‐terminal FeS cluster in yeast and aligns with Cys14 in Arabidopsis NBP35 (Figures 2a and S2), which was changed to alanine using site‐directed mutagenesis (C14A). Heterozygous *nbp35‐1*/+ plants were transformed with *NBP35*‐C14A and with wild‐type *NBP35* as a control. We used three different promoters to drive expression of the transgene: the CaMV 35S promoter; the constitutive *UBQ11* promoter; and the endogenous *NBP35* promoter. T_1_ seeds were germinated on selective medium, and plants were genotyped by polymerase chain reaction (PCR). We were unable to find any homozygous *nbp35‐1* plants with *35S:NBP35* or *35S:NBP35‐*C14A in the T_1_ or T_2_ generation (Table 1). The activity of the CaMV 35S promoter in Arabidopsis is low in female organs and absent from pollen (Wilkinson *et al*., 1997); therefore, the inability of *35S:NBP35* to complement the *nbp35‐1* knockout allele suggests an essential function of NBP35 in reproductive tissues.

**Table 1:** Segregation of *nbp35‐1* complemented with a *NBP35* or *NBP35*‐C14A transgene

*Promoter:ORF*	T_1_ a (NN:Nn:nn)	T_2_ b (NN:Nn:nn)
*35S:NBP35*	13:11:0	7:2:0 8:1:0
*35S:NBP35*‐C14A	14:7:0	7:13:0 3:6:0 5:4:0
*UBQ11:NBP35*	7:12:4	Not tested
*UBQ11:NBP35*‐C14A	9:13:2	5:4:1 3:4:1 7:2:0
*NBP35:NBP35*	8:9:1	2:5:2 9:3:1 [15]^c^:5
*NBP35:NBP35*‐C14A	8:13:0	[18]:2 [7]:2

a T_0_ plants were heterozygous NBP35/*nbp35‐1* (Nn) and T_1_ seed was pooled. Positive transformants were selected on medium with hygromycin, and genotyped for the endogenous NBP35 (N) and *nbp35‐1* (n) alleles.

b Representative genotyping results of T_2_ offspring from individual heterozygous T_1_ plants.

c Square brackets indicate the number of NN + Nn.

Expression of *NBP35* or *NBP35*‐C14A driven by the *UBQ11* promoter rescued seedling lethality of the *nbp35‐1* mutation (Table 1). The *UBQ11:NBP35* complemented plants were indistinguishable from wild‐type. In contrast, the *NBP35*‐C14A lines displayed subtle phenotypes such as enlarged leaves (Figure 2b) and ~50% shorter roots in one of the lines (Figure S3a). Protein levels of both NBP35 and NBP35‐C14A were increased ~twofold compared with wild‐type leaves, indicating that the amino acid change does not affect the stability of NBP35 (Figure 2c).

Next, we analysed a range of FeS enzymes for protein stability and/or activity. ACO protein levels were decreased in all three independent *UBQ11:NBP35*‐C14A lines but not in the *UBQ11:NBP35* lines, suggesting that impaired function of NBP35 causes protein instability of ACO due to lack of the Fe_4_S_4_ cofactor. To separate out mitochondrial and cytosolic isoforms of ACO, cell extracts were analysed using an in‐gel activity assay (Bernard *et al*., 2009). This showed that cytosolic but not mitochondrial ACO activity was decreased in the *UBQ11:NBP35*‐C14A lines (Figure 2c). The transcript levels of *ACO1*, encoding the cytosolic isoform of ACO in the leaves, were generally increased in the C14A lines compared with wild‐type (Figure S3b). The activities of AldOx and XDH, which bind two Fe_2_S_2_ clusters, were also analysed by in‐gel assays. AldOx activities were decreased as a result of NBP35‐C14A, but XDH activity was not (Figure 2c). The Fe_4_S_4_‐dependent protein ROS1 is part of a small gene family of DNA glycosylases that removes methylated cytosines. The activity of ROS1 can be determined using a PCR assay in combination with a methylation‐dependent restriction enzyme on genomic DNA sequences that are known to be demethylated by ROS1. In all three *UBQ11:NBP35*‐C14A lines the ROS1 target *XBAT34* was hypermethylated, but another target *MRD1* was not affected (Figure 2d).

**Figure 3:**
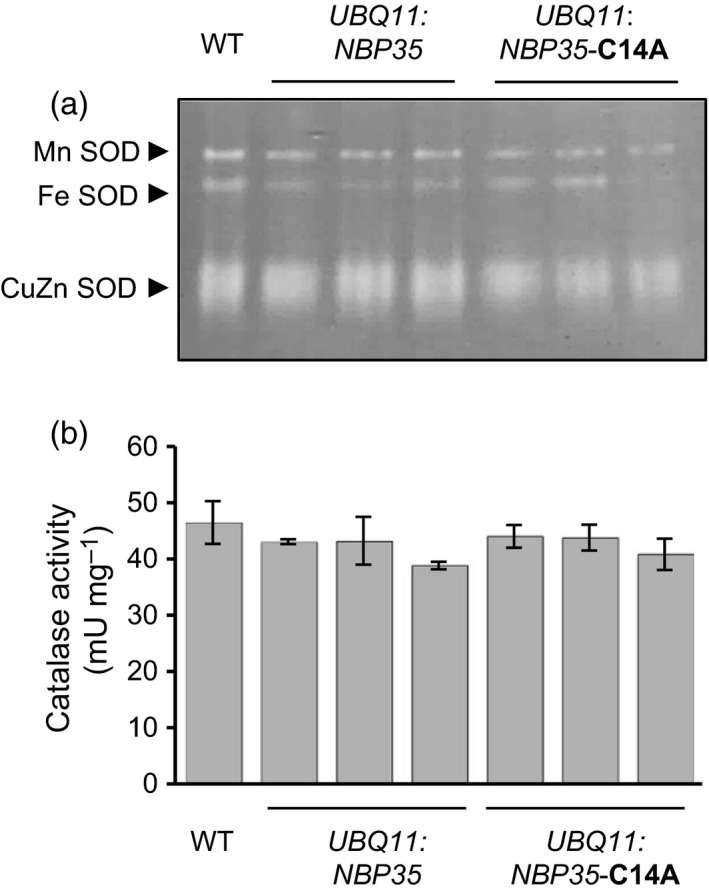
Superoxide dismutase (SOD) and catalase activities are not affected by mutation of *NBP35*. (a) SOD activities. Native polyacrylamide gel electrophoresis (PAGE) of plant extracts from wild‐type (WT), *NBP35*‐ and *NBP35*‐C14A‐expressing plants in the *nbp35‐1* background. Gels were incubated with nitroblue tetrazolium to reveal the activities as negative staining. (b) Catalase activity as measured by H_2_O_2_ consumption in a spectrophotometric assay at 230 nm.

To ensure that FeS enzyme defects were not a result of increased oxidative stress or altered metal homeostasis, the activities of superoxide dismutases (Fe, Mn and CuZn isoforms) and catalase (haem) were assessed. Neither superoxide dismutase nor catalase activities were affected in *UBQ11:NBP35*‐C14A lines (Figure 3). Taken together, these data show that impaired function of NBP35 affects some cytosolic FeS enzymes more than others, and that these changes are not due to redox imbalances.

### Low levels of NBP35‐C14A lead to severe developmental defects

**Figure 4:**
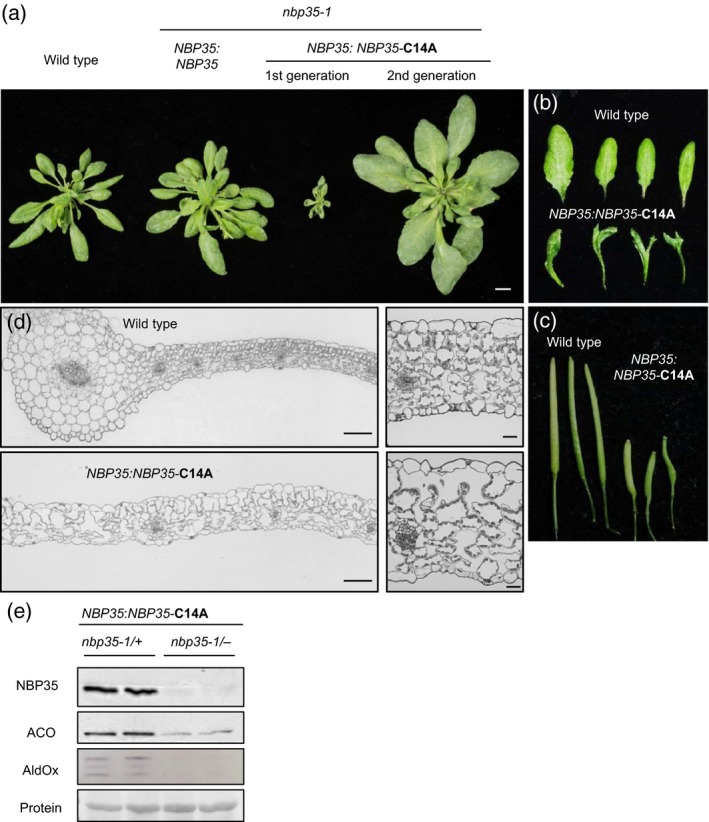
Low levels of NBP35‐C14A lead to severe developmental defects in the first generation. (a) Leaf rosette development with either *NBP35* or *NBP35‐*C14A expressed from the *NBP35* promoter in the *nbp35‐1* genetic background, as indicated. (b) Leaves and (c) siliques in the first generation of segregants carrying *NBP35:NBP35*‐C14A compared with wild‐type. (d) Cross‐sections of leaves stained with toluidine blue. Magnification × 10 (scale bar: 100 μm) in the left panels, and × 40 (scale bar: 20 μm) in the right panels. (e) NBP35 protein levels and FeS enzyme activities, as indicated.

When *NBP35* was expressed under its own promoter (−750 to −1), initially we could not find any C14A plants homozygous for *nbp35‐1* (Table 1). Extensive genotyping of offspring from *nbp35‐1/+* plants carrying the *NBP35*‐C14A transgene identified a low frequency of *nbp35‐1*/‐ segregants. Growth was extremely delayed (Figure 4a) and leaf development was abnormal, with irregular arrangement of the rosette leaves and asymmetric or even forked leaf blades (Figures 4b and S4). Further analysis of leaf anatomy showed no midvein and a loss of adaxial–abaxial polarity. The palisade parenchyma on the adaxial side was not formed in *NBP35*:*NBP35*‐C14A plants and large intercellular spaces were found throughout. Moreover, cell size was increased (Figure 4d), similar to *atm3‐1* (*starik1*; Kushnir *et al*., 2001) and *ae7* mutants (Yuan *et al*., 2010). Plants bolted after ~ 12 weeks from leaf axils. The racemes were highly branched and compact carrying short siliques with approximately four seeds (Figures 4c and S4). Interestingly, most of the phenotypes described above were not observed in the next generation (Figure 4a), only the larger leaves as seen in the *UBQ11:NBP35‐*C14A line (Figure 2b).

Protein blot analysis showed that NBP35‐C14A levels in the *nbp35‐1*/‐ segregants were strongly decreased compared with the hemizyogous *nbp35‐1/+* (Figure 4e). This may be due to regulatory sequences in the promoter or 3′UTR that were not included in the transgene. Indeed, *NBP35:NBP35* lines also had lower levels of NBP35 protein than wild‐type (Figure S4f). In *NBP35:NBP35*‐C14A plants, the FeS enzymes ACO and AldOx were much reduced in levels or activities (Figure 4e). These data suggest that NBP35 fundamentally contributes to establishing cell size and leaf polarity, most likely because FeS proteins are required for these processes.

### C14A substitution destabilized the N‐terminal Fe_4_S_4_ cluster of NBP35

**Figure 5:**
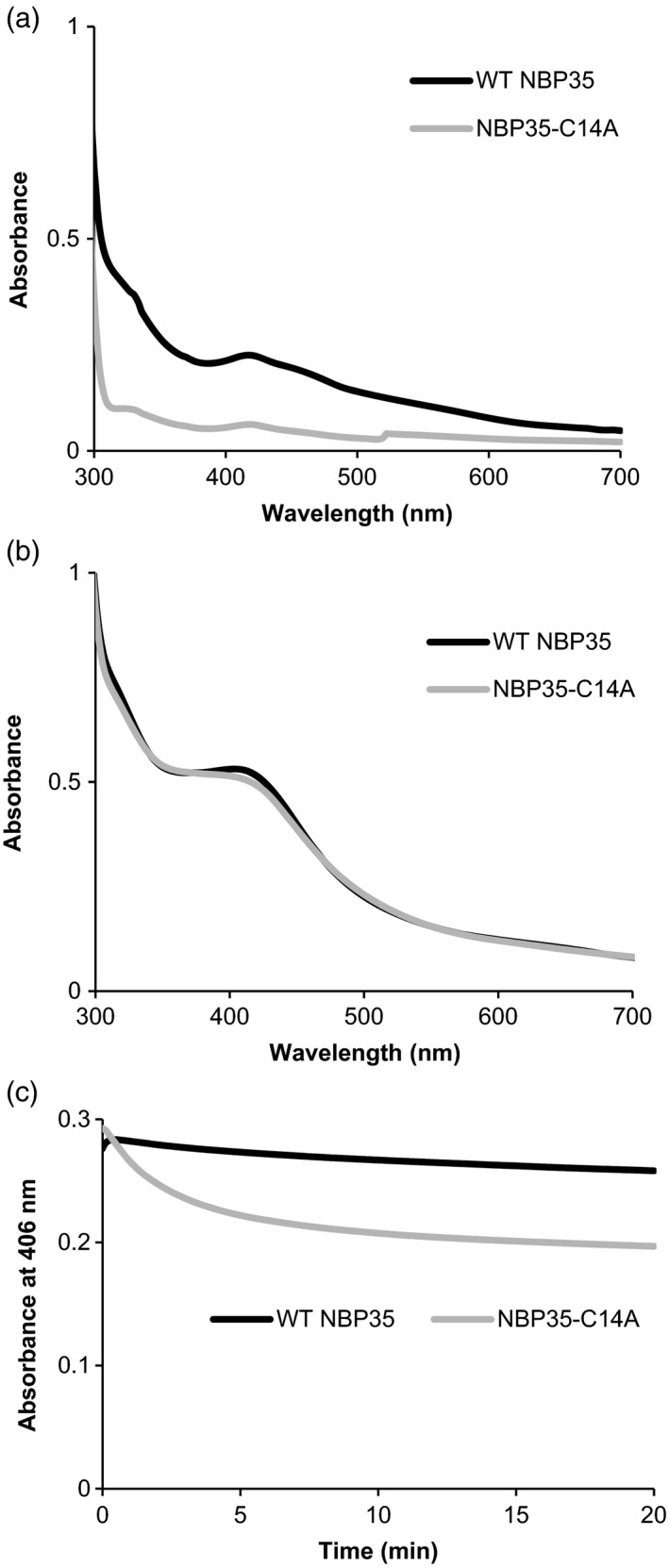
Cys14 to Ala substitution destabilizes Fe_4_S_4_ cluster binding to NBP35. (a) UV‐visible spectra of wild‐type NBP35 (black) and NBP35‐C14A (grey) as isolated from *Escherichia coli* extract. (b) UV‐visible spectra after reconstitution of FeS clusters. The broad shoulder at 400–420 nm and the feature at approximately 310 nm are indicative of Fe_4_S_4_ clusters. (c) Stability of reconstituted Fe_4_S_4_ cluster measured as the decrease in absorbance at 406 nm upon exposure to air at *t *= 0.

To investigate the effect of C14A substitution on FeS cluster binding, NBP35 and NBP35‐C14A were overproduced in *Escherichia coli* and purified, aerobically, by His‐tag affinity purification (Figure S5). The freshly isolated wild‐type protein had a pale reddish‐brown colour, whereas the C14A form was nearly colourless. UV‐visible spectroscopy of wild‐type NBP35 displayed a broad shoulder at 410 nm (Figure 5a) as previously observed, consistent with the presence of FeS clusters (Bych *et al*., 2008; Kohbushi *et al*., 2009). For the C14A protein, the spectrum was similar to wild‐type NBP35 but the absorbance at 410 nm was sixfold less, indicating changes in cluster assembly and/or stability. We noted that the shape of the ‘as isolated’ wild‐type NBP35 spectrum is reminiscent of a mixture of Fe_2_S_2_ and Fe_4_S_4_ clusters, most likely a result of cluster degradation (Tucker *et al*., 2008; Kohbushi *et al*., 2009; Crack *et al*., 2015).

Following *in vitro* anaerobic cluster reconstitution, samples of wild‐type and C14A NBP35 yielded darker, brown‐coloured solutions with UV‐visible absorbance spectra containing a broad shoulder at 400–420 nm together with a less well resolved feature at ~310 nm, indicative of the presence of Fe_4_S_4_ clusters (Figure 5b). The magnitude of the absorbance at 410 nm indicated approximately 30% of cluster occupancy. Because FeS clusters derive their optical activity from the fold of the protein to which they are ligated, circular dichroism (CD) spectroscopy can provide information about the cluster environment. The anaerobic CD spectrum of reconstituted wild‐type NBP35 displayed positive (+) features at 328, 374, 400 and 482 nm, together with negative (−) features at 444 and 550 nm. The CD spectrum of NBP35‐C14A displayed positive (+) features at 319 and 434 nm, and negative (−) features at 378 and 537 nm (see Figure S6b). Overall, the CD spectra are distinct, indicative of significant differences in the local protein environment and ligation pattern of the Fe_4_S_4_ clusters in the two proteins.

To assess the stability of the Fe_4_S_4_ clusters, wild‐type and C14A NBP35 were exposed to dissolved atmospheric oxygen, and the absorbance at 406  nm (A_406_) was followed over time. Wild‐type NBP35 displayed a 10% loss in A_406_ over 20 min (Figure 5c), with little change in its absorbance and CD spectra (Figure S6). In contrast, NBP35‐C14A displayed a 30% loss in A_406_ over the same time period, together with changes in the absorbance and CD spectra that are consistent with a mixture of Fe_2_S_2_ and Fe_4_S_4_ clusters (Figures 5c and S6). Taken together, the data demonstrate that C14A substitution destabilizes the binding of the N‐terminal Fe_4_S_4_ cluster of Arabidopsis NBP35.

### Arabidopsis NBP35 physically interacts with DRE2

NBP35 is likely to interact with upstream and downstream proteins of the CIA pathway, but such interactions have not been identified (Paul *et al*., 2015). To identify interaction partners of NBP35 in Arabidopsis, a yeast‐two‐hybrid screen was carried out using N‐terminally fused NBP35 as bait (BD‐NBP35) and an Arabidopsis seedling library of prey fragments. Of 47.8 million fragments screened there were 331 positive hits for 54 different proteins (Table S1). The results were filtered for proteins with more than one hit that were predicted or confirmed to have a cytosolic localization (http://suba3.plantenergy.uwa.edu.au/). Among the resulting eight proteins (Table 2) was NBP35, which was expected because NBP35 has been shown to form a dimer (Bych *et al*., 2008; Kohbushi *et al*., 2009) and therefore served as a positive control. For the remaining seven proteins, we considered expression patterns during Arabidopsis development (Winter *et al*., 2007). This showed that only the expression pattern of *DRE2* (*AT5G18400*) correlated with that of *NBP35* (Figure S7).

**Table 2:** Potential interacting proteins of NBP35 identified in a yeast‐two‐hybrid screen

AGI	Protein	Number of hits	Cellular localization^a^
AT1G26270	Putative phosphatidylinositol 4‐kinase type 2‐beta	5	Cytosol
AT1G60710	Probable aldo‐keto reductase 4 (ATB2)	12	Cytosol
AT5G18400	Anamorsin homologue (DRE2)	3	Cytosol, plasma membrane
AT3G04120	Glyceraldehyde‐3‐phosphate dehydrogenase C subunit (GAPC1)	3	Cytosol
AT3G16460	Jacalin‐like lectin domain‐containing protein	2	Cytosol
AT5G50960	Nucleotide binding protein 35 (NBP35)	2	Cytosol
AT1G73010	Inorganic pyrophosphatase 1 (PS2)	17	Cytosol
AT5G10540	Zincin‐like metalloproteases family protein (TOP2)	21	Cytosol

47.8 million clones from an Arabidopsis seedling cDNA library were screened, resulting in 331 hits for 54 different gene fragments. Those coding sequences with ≥ 2 hits for proteins with a cytosolic localization are listed here. The full list can be found in Table S1.

a Based on http://suba3.plantenergy.uwa.edu.au/.

**Figure 6:**
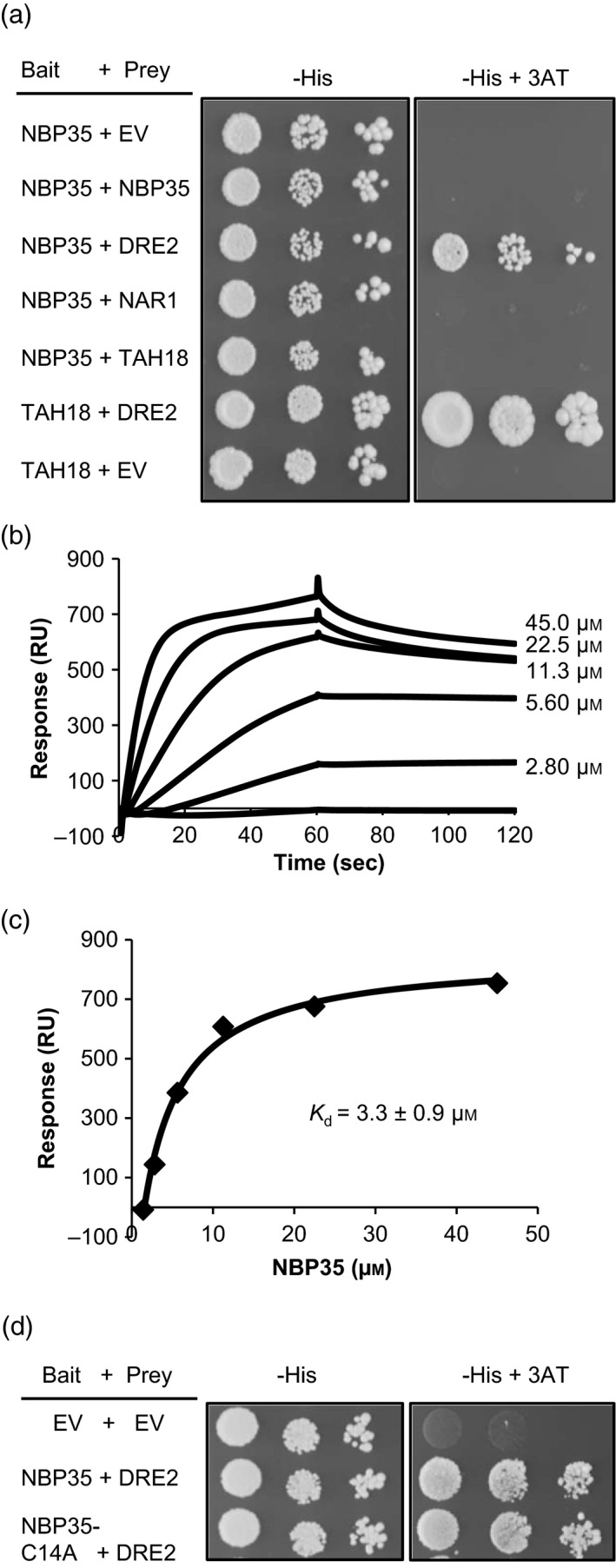
NBP35 interacts with DRE2. (a) Yeast‐two‐hybrid analysis of Arabidopsis NBP35 BD‐fusion protein with either empty vector (EV), NBP35, DRE2, NAR1 or TAH18 AD‐fusion proteins. The TAH18‐DRE2 interaction served as a positive control. Background growth on dropout histidine medium (‐His) was inhibited using 100 mm of the competitive inhibitor 3‐amino‐1,2,4‐triazole (3‐AT). (b) Surface Plasmon Resonance (SPR) sensorgrams showing binding of NBP35 to immobilized DRE2. (c) Response units from (b) were plotted against NBP35 concentrations to calculate the affinity of NBP35 for DRE2. (d) Yeast‐two‐hybrid analysis as in (a) except with NBP35‐C14A.

To confirm the protein interaction between NBP35 and DRE2, full‐length NBP35 was used as bait and full‐length DRE2 was used as prey with a different yeast‐two‐hybrid system (INVITROGEN ProQuest kit). DRE2 could not be used as bait because this auto‐activated the reporter genes. Yeast growth on selective medium demonstrated interaction between NBP35 and DRE2 (Figures 6a and S8). The strength of the NBP35–DRE2 protein interaction was compared with the previously reported DRE2–TAH18 physical interaction (Varadarajan *et al*., 2010) using 3‐amino‐1,2,4‐triazole (3‐AT), a competitive inhibitor of the protein product of the *HIS3* gene. Growth of yeast depending on the DRE2–TAH18 protein interaction was much more robust than for the NBP35–DRE2 combination, suggesting that the binding affinity of DRE2 for NBP35 is relatively weak compared with TAH18 (Figure 6a).

The interaction between NBP35 and DRE2 was further tested using Surface Plasmon Resonance (SPR). Strep‐tagged DRE2 was immobilized on a Streptavidin chip and the binding of increasing concentrations of purified NBP35 (0–45 μm) was recorded in a multi‐cycle experiment. The positive response during injection of NBP35 (Figure 6b, first 60 s) showed that NBP35 bound to DRE2. The dissociation of NBP35 was slow, suggesting a relatively strong binding once NBP35 and DRE2 interact. Fitting the response to the concentrations of NBP35 gave an affinity of 3.3 ± 0.9 μm (Figure 6c). Taken together, the results from yeast‐two‐hybrid and SPR experiments show a specific protein interaction between NBP35 and DRE2.

We also investigated protein interaction between NBP35 and NAR1 from Arabidopsis, a protein downstream in the CIA pathway. Yeast‐two‐hybrid analysis with NBP35 as prey and NAR1 as bait did not show an interaction (Figure 6a). It is possible that the observed interactions between Arabidopsis CIA proteins could be affected by the presence of yeast homologues, but we think this is unlikely because Arabidopsis *NBP35* cannot complement yeast *nbp35* mutants (Bych *et al*., 2008; Kohbushi *et al*., 2009) and Arabidopsis *DRE*2 cannot complement a yeast *dre2* mutant unless co‐expressed with Arabidopsis *TAH18* (Bernard *et al*., 2013).

Because C14A substitution in NBP35 decreases the activity of FeS enzymes, we tested the effect of this substitution in the yeast‐two‐hybrid assay. We found that interaction between NBP35‐C14A and DRE2 was similar to the interaction with wild‐type NBP35 (Figure 6d). This indicates that cluster binding to the N‐terminal domain is not required for the interaction.

## Discussion

Proteins of the CIA pathway are highly conserved in eukaryotes (Figure S9), but they remain relatively understudied. Both the essential nature of most *CIA* genes and low levels of expression are likely to contribute to their elusiveness. In plants, several *CIA* genes have recently been identified in mutant screens for gene silencing by DNA methylation: *AE7*/*CIA2*,* DRE2*,* NAR1* and *MET18* (Yuan *et al*., 2010; Nakamura *et al*., 2013; Buzas *et al*., 2014; Duan *et al*., 2015; Wang *et al*., 2016). Because a comprehensive phenotypic analysis has only been carried out for *AE7* (Luo *et al*., 2012), we undertook a study to define the role of Arabidopsis NBP35 in the maturation of FeS enzymes and in plant development.

Depletion of NBP35 to less than 30% of wild‐type protein levels did not result in any obvious phenotype or in decreased FeS enzyme activities in the leaves (Figures 1b and c, and S1). Either NBP35 protein levels are in excess in the vegetative stage, or the effect on FeS enzymes is indirect, via another process important for FeS cluster assembly. The homologous INDH protein in mitochondria is also thought to play an indirect role in the assembly of the eight FeS clusters of complex I, although its precise function has not been identified yet (Wydro *et al*., 2013). In contrast to the RNAi lines, stable expression of a mutated version of *NBP35* showed a clear decrease in a number of cytosolic FeS enzymes, namely ACO, AldOx and, to some extent, the DNA glycosylase ROS1 (Figure 2c and d). The relatively mild nature of the C14A substitution may explain that not all FeS enzymes are equally affected, with XDH not affected at all. At the same time, the results indicate a certain priority in the maturation of FeS proteins, also observed in *ae7* Arabidopsis mutants and in human cells depleted of the NBP35 homologue (Stehling *et al*., 2008).

Low levels of NBP35‐C14A resulted in severe developmental defects, which were evidently suppressed by overexpression of NBP35‐C14A using the *UBQ11* promoter (Figures 3a–c and S4). The asymmetric leaves lacked the differentiated cell layers that define the upper (adaxial) and lower (abaxial) side of the leaf, the palisade and spongy parenchyma, respectively. Moreover, cells were enlarged in the mutants. The change in anatomy and cell size indicates a problem with cell division and establishment of tissue polarity. Interestingly, this has also been observed in nbp35 (*nubp1*) mutants in mouse during lung development and ciliogenesis (Schnatwinkel and Niswander, 2012; Kypri *et al*., 2014; Ververis *et al*., 2016). Identification of a protein interaction between Nubp1 and the kinesin‐like protein (KIFC5A) involved in bipolar spindle assembly further implicated Nubp1 in the cell cycle (Christodoulou *et al*., 2006). Alternatively, a defect in ribosome function could also lead to aberrant leaf development as shown for a mutant in *RNase L inhibitor 2* (*RLI2*) in *Cardamine hirsuta*, a plant species related to Arabidopsis (Kougioumoutzi *et al*., 2013). RLI is highly conserved in eukaryotes and Archaea, including its two Fe_2_S_2_ cluster binding motifs, and is essential for ribosome assembly. It is interesting to note that many of the CIA proteins have been identified in screens for ribosome mutants, including the *DRE2* gene in yeast.

While the first generation of *NBP35:NBP35*‐C14A plants had severe developmental phenotypes, the few seeds they produced developed normally except that the plants were significantly bigger in size. A possible explanation of this unusual observation is that the limited function of NBP35 causes genome instability, as found in *ae7* mutants (Luo *et al*., 2012), which could disrupt meiosis and prevent fertilization. The few seeds that survive may have one or more suppressor mutations, enabling vigorous growth in the next generation. Whole genome sequencing of first‐ and second‐generation plants could be used to identify such mutations.

In yeast, mammals and other metazoa NBP35 has been shown to interact consistently with CFD1/Nubp2. No interactions between NBP35 and other CIA proteins have been found so far, suggesting that such interactions may be transient, reliant on FeS occupancy and/or require binding of multiple proteins. The absence of CFD1 in plants may be the reason why we were able to detect DRE2 as an interaction partner of NBP35 (Figures 6 and S8; Table S1). DRE2 has already been shown to interact with the flavoprotein TAH18 (Varadarajan *et al*., 2010), forming an electron transport chain in an early step required for FeS protein biogenesis (Netz *et al*., 2010; Bernard *et al*., 2013). It will be interesting to investigate if NBP35 competes with TAH18 for binding to DRE2, or whether the NBP35–DRE2 interaction is more prevalent under oxidative stress, when yeast Tah18 relocalizes to the mitochondria without Dre2 (Vernis *et al*., 2009).

Mutation of Cys14 in the N‐terminus destabilized the cluster and altered the protein folding (Figures 5 and S6), but this did not affect the DRE2–NBP35 interaction (Figure 6d). These findings could suggest that the N‐terminal domain of NBP35 is not the main determinant in the interaction with DRE2. If that is the case, it would follow that electrons are transferred between DRE2 and the C‐terminal Fe_4_S_4_ cluster of NBP35. As yet, there is no experimental evidence for electron transfer between DRE2 and NBP35, although this is suggested in schematics of the CIA pathway. To probe exactly which domains of DRE2 and NBP35 interact, further yeast‐two‐hybrid experiments need to be carried out.

In conclusion, we have shown that Arabidopsis NBP35 interacts with DRE2 and that NBP35 is required for the activity of downstream cytosolic FeS enzymes. Further studies are required to determine how NBP35 transfers FeS clusters to downstream targets and how this links to the observed developmental phenotypes of *nbp35* mutants.

## Experimental procedures

### Plant material and growth

*Arabidopsis thaliana* ecotype Columbia (Col‐0) plants were used as the wild‐type. The T‐DNA insertion lines *nbp35‐1* (SALK_056204) and *nbp35‐3* (SALK_054678) were from Nottingham Arabidopsis Stock Centre and previously described (Bych *et al*., 2008; Kohbushi *et al*., 2009; Nakamura *et al*., 2013). The T‐DNA insertion sites were confirmed by sequencing: +1 in exon 2 for *nbp35‐1*, and −49 in the 5′UTR for *nbp35‐3*. Seeds were sterilized using chlorine gas, vernalized for 2 days at 4°C and germinated on half‐strength Murashige and Skoog medium (MS) with 0.8% (w/v) agar. Seedlings were transplanted to compost after 2 weeks. All plant growth was under long‐day conditions, 16 h light at 22°C, 8 h dark at 20°C, unless otherwise stated, with 80% humidity and a light intensity of 140–200 μmol m^−2^ s^−1^. For growth under short‐day conditions, seeds were sown directly onto soil and grown under 8 h light/16 h dark cycles.

### Molecular cloning, site‐directed mutagenesis and plant transformation

For downregulation of *NBP35* expression by RNAi, 121 nucleotides of the *NBP35* coding sequence (*AT5G50960*, c.517–627) were placed in sense and antisense orientation under the control of the ethanol‐inducible *alcR‐alcA* gene expression system (Roslan *et al*., 2001). Homozygous plants from the T3 generation were sprayed with 2% (v/v) ethanol from day 14, every other day, and tissue was harvested between 4 and 5 weeks.

For amino acid substitution, the *NBP35* coding sequence was PCR‐amplified from cDNA and cloned behind the CaMV 35S promoter using NcoI and XbaI in a pUC‐derived cloning plasmid. Cys14 to Ala substitution in NBP35 was introduced by site‐directed mutagenesis using Phusion polymerase as per manufacturer's instructions (NEB M0530) with primers EB1 and EB2 (see Table S2 for primer sequences). The *NBP35* promoter and 5′UTR, starting at 750 nucleotides upstream of the start codon, were amplified using primers EB13 and EB15 containing restriction sites AscI and NcoI, respectively. The DNA fragment was digested and inserted in the cloning plasmid to replace the CaMV 35S promoter. Similarly, the *UBQ11* (*AT4G05050*) promoter was amplified by PCR with primers EB16 and EB17 containing restriction sites AscI and XhoI.

The *promoter:NBP35* and *promoter:NBP35*‐C14A sequences were cut from the cloning plasmid with AscI and PacI restriction enzymes, and ligated into the binary vector pBIN containing a hygromycin‐selective marker, resulting in *35S:NBP35*,* NBP35:NBP35*,* UBQ11:NBP35*,* 35S:NBP35‐C14A*,* NBP35:NBP35*‐C14A and *UBQ11:NBP35*‐C14A constructs. Heterozygous *nbp35‐1*/+ plants were transformed by the floral‐dip method using the *Agrobacterium tumefaciens* strain GV3101. Successfully transformed plants were selected on ½ MS with 0.8% (w/v) agar plus 25 μg μL^−1^ hygromycin. Plants were genotyped for the presence of the *NBP35* transgene using exon primers EB21 and EB22 that span an intron *of NBP35*. Zygosity of the *nbp35‐1* allele was determined using the primers EB77 (LBb1.3) in the T‐DNA and EB32 in the *NBP35* gene.

### Protein blot analysis

Protein extracts were separated by sodium dodecyl sulphate (SDS)–polyacrylamide gel electrophoresis (PAGE) and transferred under semi‐dry conditions to nitrocellulose membrane for immunolabelling. Ponceau‐S staining of the membranes was used to confirm equal protein loading and successful transfer. Polyclonal antibodies against Arabidopsis NBP35 and ACO were as previously described (Bych *et al*., 2008; Bernard *et al*., 2009).

### Enzyme assays

In‐gel assays for AldOx, XDH and ACO were as previously reported (Bernard *et al*., 2009). Catalase activity was measured using a spectroscopic assay for H_2_O_2_ (Beers and Sizer, 1952). Superoxide dismutase activity was measured according to Chu *et al*. (2005). The McrBC‐PCR assay was performed as previously described (Luo *et al*., 2012) using specific primers for the *XBAT34* and *MDR1* loci (Table S2).

### Microscopy

Leaf material was fixed with a solution of 3.7% (w/v) formaldehyde, 5% (v/v) acetic acid, 50% (v/v) ethanol and embedded in LR white. Transverse sections (0.5 μm) were stained with toluidine blue and viewed on a LEICA DM6000 light microscope. The objectives used were ×10/0.4 air, ×20/0.7 air or ×40/0.85 air, and overview images were collected by taking a series of partially overlapping images with a LEICA DFC420C colour camera, which were merged using LEICA LAS‐AF software.

### Protein interaction assays

The ULTImate Y2H screen was carried out by HYBRIGENICS SERVICES (Paris, France) using an *A. thaliana* seedling cDNA library (ecotype Columbia, 1‐week‐old seedlings). NBP35 was used as bait and N‐terminally fused to the DNA‐binding domain. To test binary interactions the INVITROGEN ProQuest Two‐Hybrid System with Gateway Technology was used. The coding sequences of Arabidopsis *NBP35*,* DRE2, TAH18* and *NAR1* were N‐terminally fused to the DNA‐binding domain (BD) and/or DNA‐activation domain (AD) as indicated. The BD‐DRE2 protein auto‐activated transcription and was therefore not used.

Surface Plasmon Resonance was performed using a GE HEALTHCARE (Little Chalfont, UK) Biacore T200 instrument with a Streptavidin sensor chip. After washing with 1 m NaCl, 50 mm NaOH at a flow rate of 10 μL min^−1^ to remove any unconjugated Streptavidin, *E. coli* cell extract containing Strep‐tagged DRE2 (Bernard *et al*., 2013) was allowed to flow over the surface at a flow rate of 30 μL min^−1^ for 30 sec. A response of approximately 1700 response units was obtained, indicating that DRE2 was bound to the chip. Purified NBP35 was diluted in running buffer (20 mm Tris‐HCl pH 8, 0.15 m NaCl, 5% glycerol) and injected over immobilized DRE2‐Strep and over a control flow cell without DRE2‐Strep. Multi‐cycle binding experiments were performed at 25°C at a flow rate of 30 μL min^−1^. Each concentration of NBP35 was injected for 60 sec and then switched to buffer flow to monitor the dissociation for 60 sec. The chip surface was regenerated by one 30‐sec injection of 1 m NaCl, 10 mm NaOH followed by washing with running buffer for 60 sec. Non‐selective binding to the chip surface was accounted for by subtracting the response in the control flow cell. The average equilibrium response taken 4 sec before the end of the injection was plotted against NBP35 concentrations using the Affinity Fit option from the Biacore T200 evaluation software v2.0 assuming a 1:1 binding model.

### Reconstitution of FeS clusters, and UV‐visible absorbance and CD spectroscopies

The full‐length Arabidopsis *NBP35* coding sequence was cloned into the pET15b expression vector (NOVAGEN) in‐frame with an N‐terminal His tag (Bych *et al*., 2008). Site‐directed mutagenesis was used to introduce a Cys to Ala substitution at position 14. Protein overexpression and affinity purification were carried out following the manufacturer's instructions using a His‐Trap HP column (GE HEALTHCARE) on an ÄKTA Pure system with a linear gradient of imidazole. NBP35 (wild‐type or C14A)‐containing fractions were immediately desalted with 20 mm Tris‐HCl pH 8, 150 mm NaCl and 5% (v/v) glycerol (wash buffer) using a PD10 column (GE HEALTHCARE). NifS‐catalysed *in vitro* cluster reconstitution was used to assemble FeS clusters on NBP35, as previously described (Crack *et al*., 2014). Briefly, NBP35 samples containing 70–90 μm protein in approximately 3.5 mL were treated with 3.75 mm dithiothreitol at 37°C for 15 min, followed by addition of 1.5 mm L‐Cys, 113 nm NifS and a sixfold molar excess of ferrous ammonium sulphate, and incubated at 37°C for 100 min. Reconstituted protein was then separated from low molecular weight species via a PD10 desalting column previously equilibrated with wash buffer.

UV‐visible absorbance measurements were made with a JASCO V550 spectrophotometer. CD spectra were measured with a JASCO J810 spectropolarimeter. An extinction of e_406 nm_ ≈ 16 000 m ^−1^ cm^−1^ (Sweeney and Rabinowitz, 1980) was used to estimate the amount of Fe_4_S_4_ cluster present in NBP35 samples. Cluster stability was investigated under pseudo‐first order conditions (~120 μm O_2_) by combining varying aliquots of aerobic and anaerobic wash buffer (2 mL total volume) with NBP35 (~16 μm Fe_4_S_4_). Changes in the absorbance at 406 nm were used to track cluster stability.

## Accession numbers

NBP35 (AT5G50960), DRE2/CIAPIN1 (AT5G18400), TAH18/ATR3 (AT3G02280), NAR1 (AT4G16440).

## Supporting information


**Figure S1.** Quantification of NPB35 protein levels in knockdown lines.
**Figure S2.** Alignment of *Saccharomyces cerevisiae* and *Arabidopsis thaliana* NBP35.
**Figure S3.** Root growth and transcript levels of FeS proteins in *NBP35*‐C14A.
**Figure S4.** Developmental defects in plants expressing *NBP35*:*NBP35*‐C14A.
**Figure S5.** Expression and purification of NBP35 and NBP35‐C14A.
**Figure S6.** Spectroscopy of reconstituted NBP35 and NBP35‐C14A.
**Figure S7.** *NBP35* and *DRE2* gene expression during development.
**Figure S8.** Confirmation of yeast‐two‐hybrid interactions identified in the screen.
**Figure S9.** Model of the CIA pathway in plants.

**Table S1.** Yeast‐two‐hybrid interactions of Arabidopsis NBP35 screened against a fragment library of seedling cDNA
**Table S2.** List of primers.
