# Supplementary material for: NBP35 interacts with DRE2 in the maturation of cytosolic iron‐sulphur proteins in Arabidopsis thaliana
Source: Plant J. 2017 Feb 3;89(3):590–600. doi: 10.1111/tpj.13409 (PMC5324674; doi:10.1111/tpj.13409)

Figure S1. Quantification of NPB35 protein levels in knockdown lines.

Protein samples were separated by SDS-PAGE, transferred to nitrocellulose, and labelled with antibodies specific to Arabidopsis NBP35 (Bych *et al* (2008) *J. Biol. Chem.*, 283, 35797-804). The signal was developed with secondary horse-radish peroxidase conjugated antibodies, ECL reagents and exposure to film. The intensity of the bands was quantified with ImageJ software and the values in the tables represent arbitrary units for the relative density of the peaks. The prominent band of Rubisco large subunit (RbcL) on the Ponceau S-stained nitrocellulose blot was used to adjust for differences in protein loading and protein transfer.

(a) RNAi lines

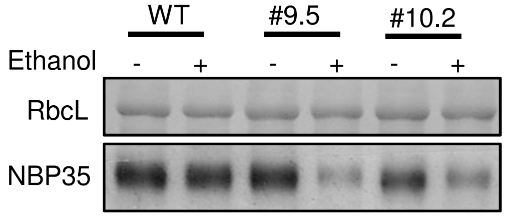

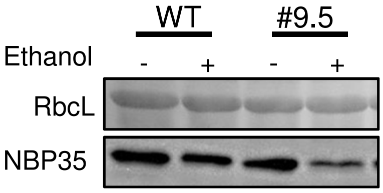

	WT -	WT +	RNAi *nbp35* -	RNAi *nbp35* +	RNAi *nbp35* -	RNAi *nbp35* +
RbcL	4706	3897	4912	4177	5266	5119
NBP35	4016	3078	3589	908	2908	1279
Normalised	4016	3716	3438	1023	2599	1175
% of WT	**100**	**93**	**86**	**25**	**65**	**29**

WT -	WT +	RNAi *nbp35* -	RNAi *nbp35* +
2196	1609	1508	1855
4240	2274	2918	1214
4240	3104	4250	1437
**100**	**73**	**100**	**34**

(b) *nbp35-3*

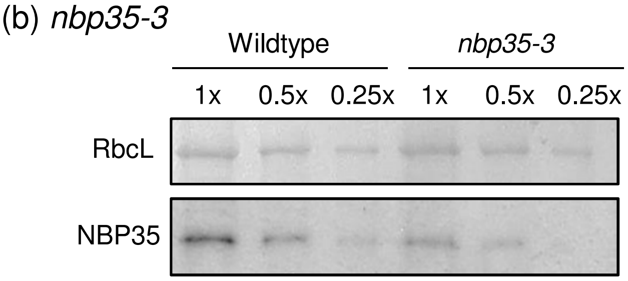

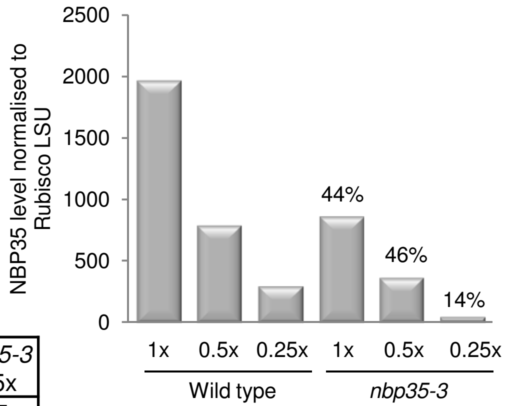

	WT 1x	WT 0.5x	WT 0.25x	*nbp35-3* 1x	*nbp35-3* 0.5x	*nbp35-3* 0.25x
RbcL	1932	876	364	1979	782	427
NBP35	1965	786	293	883	321	48
Normalised				862	359	41
% of WT				**44**	**46**	**14**

	WT	*nbp35-3*
RbcL	348	307
NBP35	334	130
Normalised		115
% of WT		**35**

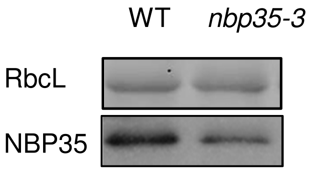

	WT	*nbp35-3*
RbcL	2580	2351
NBP35	3233	2170
Normalised		1977
% of WT		**61**

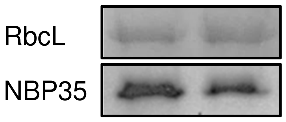

Figure S2. Alignment of *S.cerevisiae* and *A. thaliana* NBP35.

The multiple sequence alignment was made with the T-COFFEE server at http://www.ebi.ac.uk/Tools/msa/tcoffee/

```
AtNBP35         MENGDIPEDANEHCPGPQSESAGKSDSCAGCPNQEACATAPKGPDPDLVA
ScNbp35         MTH--VNDVAYNHCG---SDMAGKSDACGGCANK--C---SKGDDTDN--
                  * :  : : * :**    *: *****:*.**.*:  *   .** *.*

AtNBP35         IAERMSTVKHKILVLSGKGGVGKSTFSAQLSFALAGMDHQVGLMDIDICG
ScNbp35         -------SGHK---VSGKGGVGKST--AAMSWASA---DDVGAMD--DCG
                         **   :**********  * :*:* *   .:** **   **

AtNBP35         PSIPKMLGLEGQEIHQSNLGWSPVYVEDNLGVMSIGFMLPNSDEAVIWRG
ScNbp35         SHM-------GCKTVHSNSGWT-VYVTDNAT-MS--YM----DD-SAWRG
                . :       * :  :** **: *** **   **  :*    *:   ***

AtNBP35         PRKNGLIKQFLKDVYWGEIDYLVVDAPPGTSDEHISIVQYLLPTGIDGAI
ScNbp35         SKKNK-----KKDVDWDK-DYV--D--TGTSDHSN---KYM-RS-GDGAV
                .:**       *** *.: **:  *  .****.     :*:  :  ***:

AtNBP35         IVTTPQEVSLIDVRKEVSFCKKVGVPVLGVVENMSGLSQPLKDVKFMKLA
ScNbp35         VTT------VADVRKD---CKKAGNGV-----NMSGV-------------
                       :.*      : ****:   ***.*  *     ****:

AtNBP35         TETGSSINVTEDVIACLRKNAPELLDIVACSEVFDSSGGGAERMCREMGV
ScNbp35         ---------------------------CNCKGSKATTGGGA---CKGKG-
                                              *.    ::****   *:  *

AtNBP35         PFLGKVPMDPQLCKAAEQGKSCFEDNKCLISAPALKSIIQKVVPSTVMTE
ScNbp35         --------------SVDRGKSCDMGSDNYDSAS---SAVNVVARDAVGDV
                                :.::****  ...   **.   * :: *. .:*
```

Figure S3. Root growth and transcript levels of FeS proteins in *NBP35*-C14A

(a) Root growth of 11-day-old *nbp35*-1 expressing *NBP35* or *NBP35*-C14A from the *UBQ11* promoter.

(b) Transcript levels of *NBP35*, the cytosolic aconitase isogene (*ACO1*) and the DNA glycosylase *ROS1* were analysed by semi-quantitative RT-PCR. RNA samples were prepared from leaves from the same plant material in Figure 2b and c.

(a)

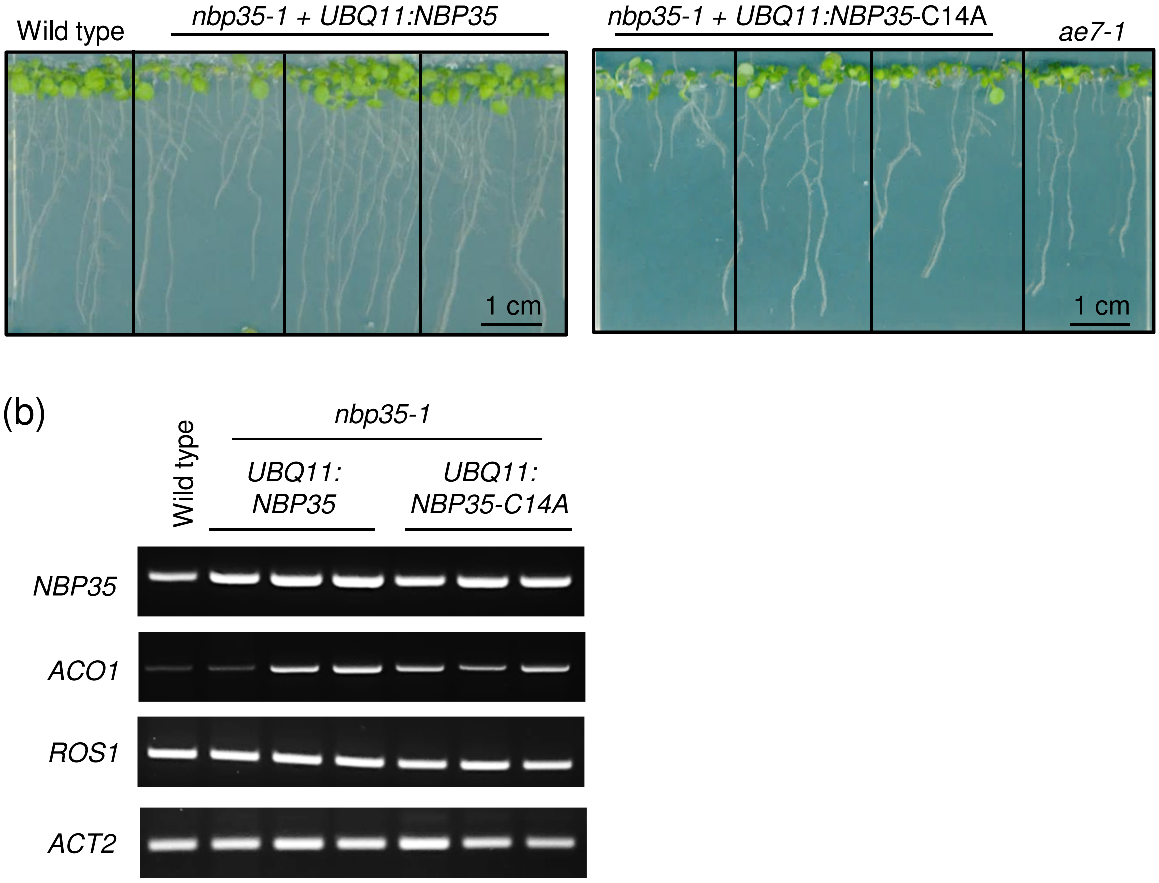

Figure S4. Developmental defects in plants expressing *NBP35*:*NBP35*-C14A.

(a) Four-week-old plants expressing *NBP35:NBP35*-C14A segregating from a heterozygous *nbp35-1*/+ parent. PCR analysis showed that the two plants on the left are heterozygous (*nbp35-1*/+) and the two plants on the right are homozygous for the *nbp35-1* knockout allele.
(b) Six-week-old wild type plants (WT) and plants expressing *NBP35*:*NBP35*-C14A in the *nbp35-1*/- mutant.
(c-e) *nbp35-1*/- plants expressing *NBP35:NBP35*-C14A
(f) Protein blot analysis on leaf extracts from the indicated plant lines with antibodies against NBP35 and aconitase (ACO)

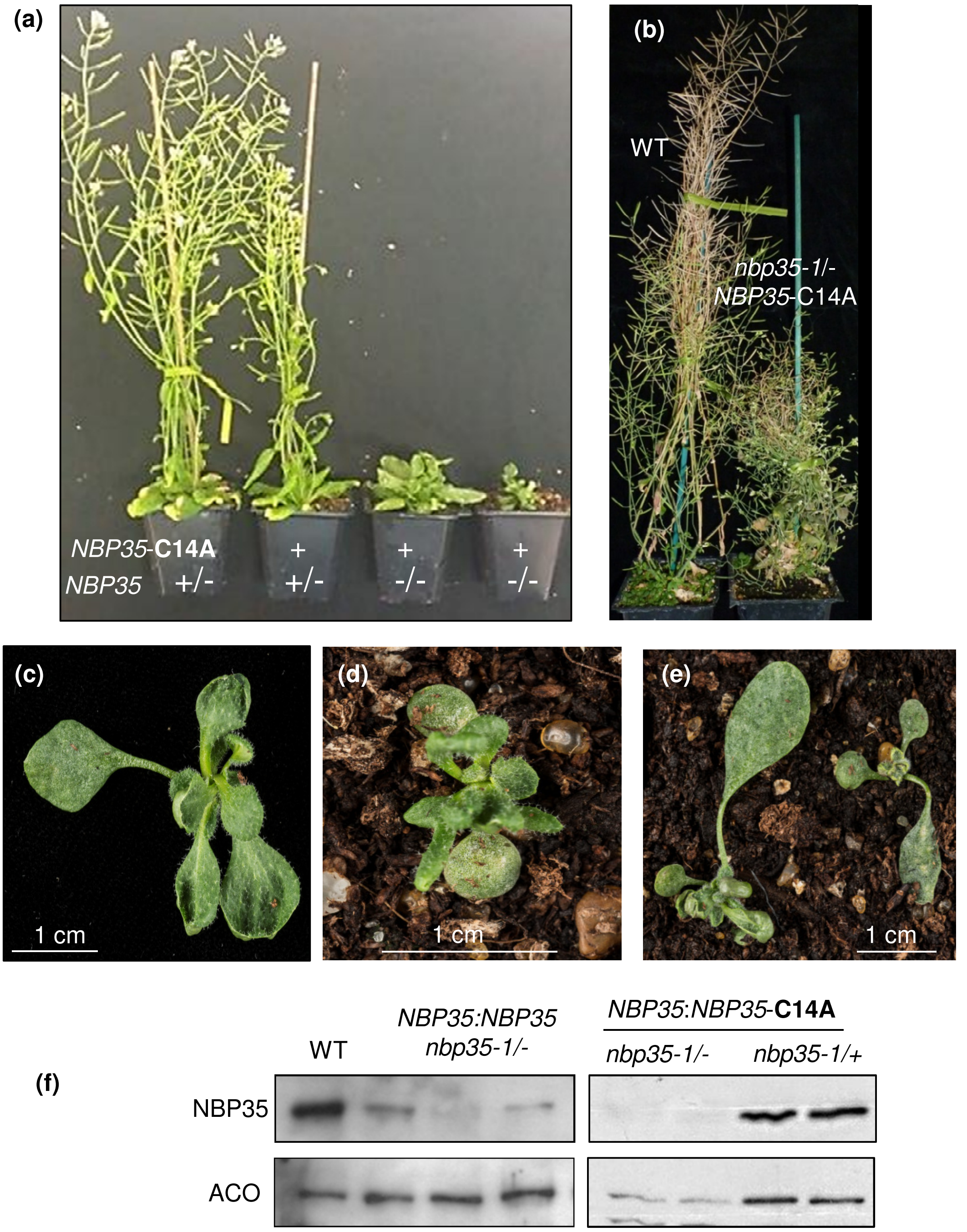

Figure S5. Expression and purification of NBP35 and NBP35-C14A.

Purification of NBP35 (a) and NBP35-C14A (b) from *E. coli*. Aliquots of total extract (T), the soluble protein fraction (S), flow through from the Ni-NTA column (FT), washes (W) and the eluting peak were run on SDS-PAGE and stained with Coomassie InstantBlue (EXPEDEON).

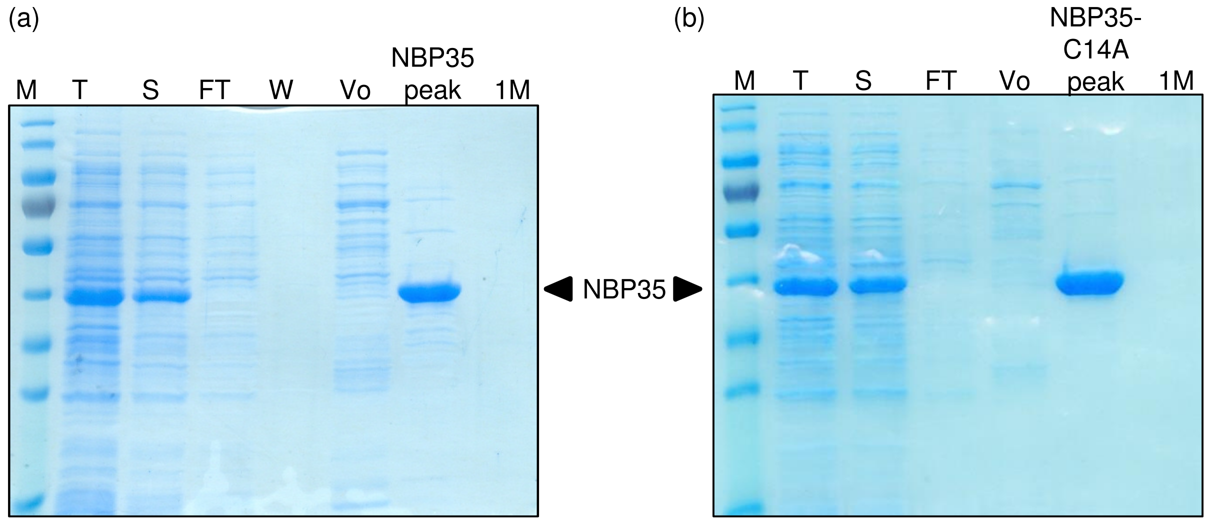

Figure S6. Spectroscopy of reconstituted NBP35 and NBP35-C14A

(a) UV-visible spectrum after addition of buffer containing dissolved atmospheric oxygen. (b) Circular dichroism (CD) of wild-type and C14A forms of NBP35 after reconstitution and (c) after 20 min exposure to air.

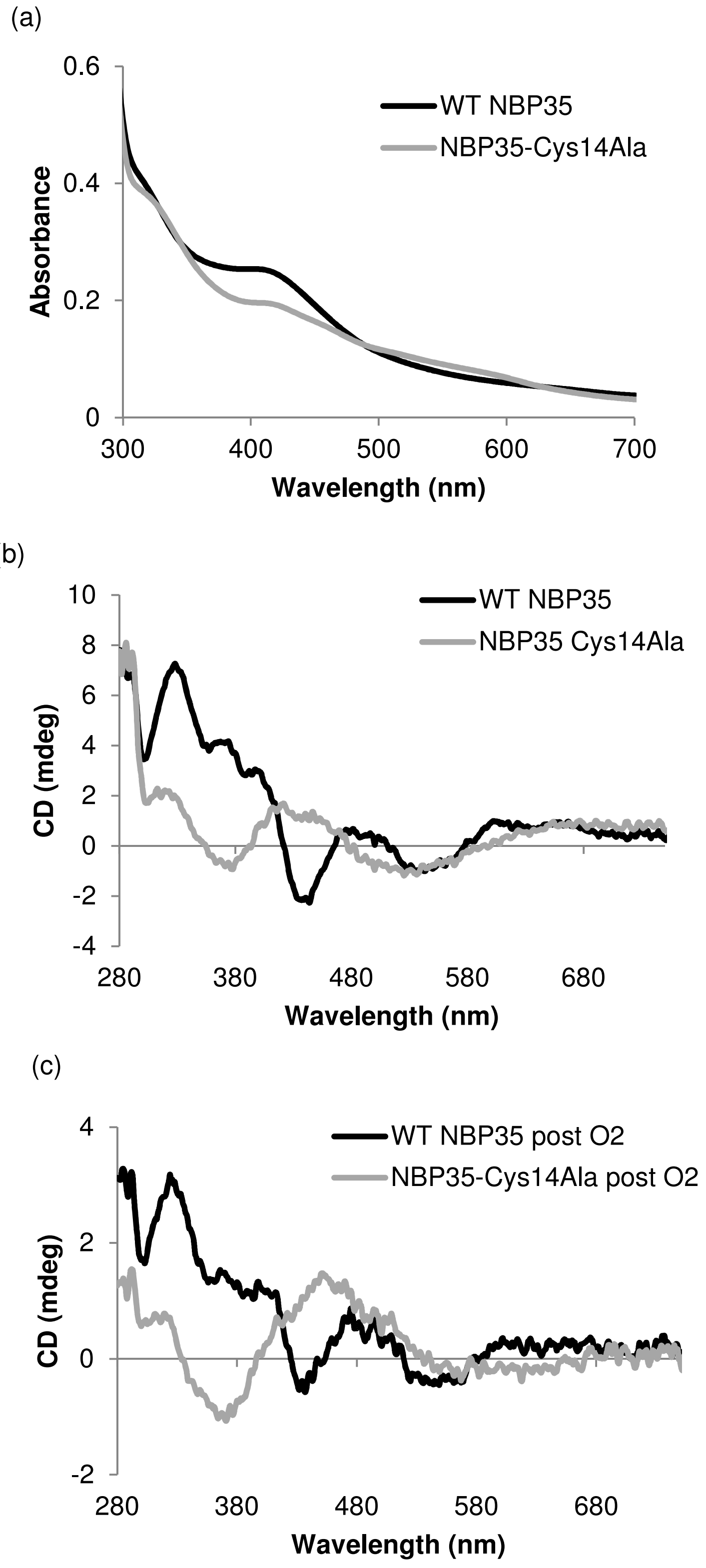

Figure S7. *NBP35* and *DRE2* gene expression during development.

The Arabidopsis eFP browser was used to visualise data for gene expression during Arabidopsis development. These data were created by Winter *et a*l (2007) *PLoS One.,* 2, 1-12. Screenshots of the developmental map show expression in different tissues and this was used to determine whether proteins identified in the yeast-two hybrid screen were likely to co-occur. *NBP35* and *DRE2* have similar expression patterns whereas *TOP2* for example, the candidate with 21 hits in the yeast-two hybrid screen does not. *TOP2* has higher expression throughout different tissues compared to *NBP35* except in dry seeds where expression of *NBP35* is very high and expression of *TOP2* is very low. Low expression of *NBP35* in all reproductive tissues differs from *TOP2* which has high expression specifically in the carpels.

NBP35

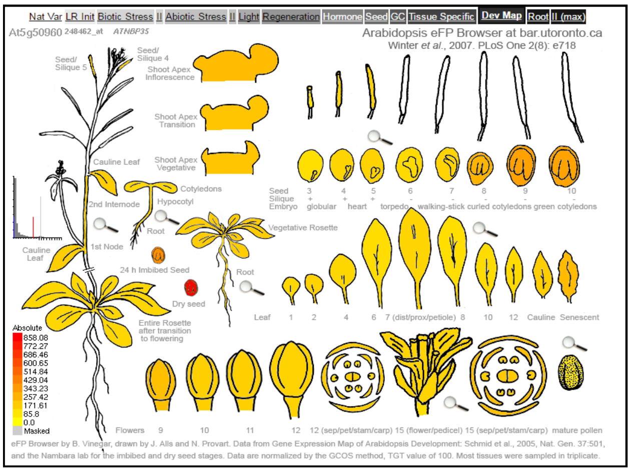

DRE2

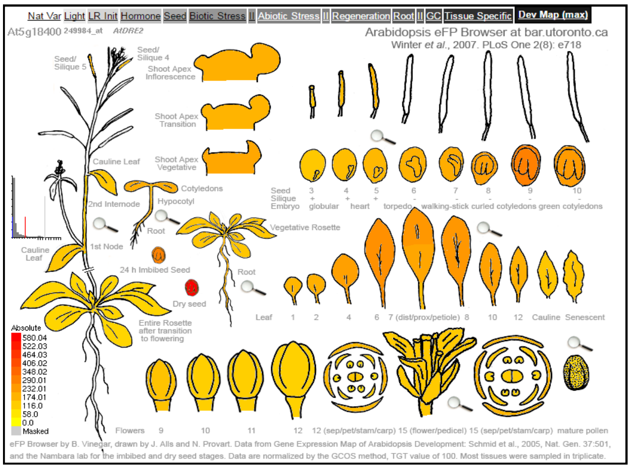

TOP2

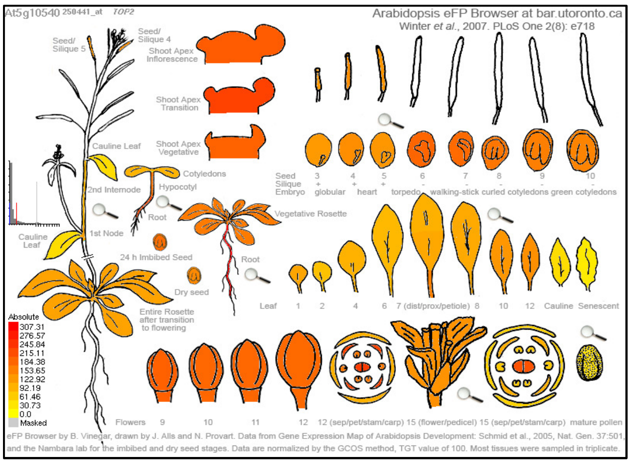

Figure S8. Confirmation of Yeast-2 Hybrid interactions identified in the screen.

Yeast strain MaV203 was transformed with pDEST32 (BD) and pDEST22 (AD) fused to the indicated genes or as empty vectors (EV) and grown for 3 days on amino acid dropout medium lacking Leu and Trp. Independent colonies were picked and restreaked onto dropout Leu,Trp medium and grown 2-3 days before replica plating onto dropout Leu, Trp, His medium or –Leu, Trp, His + 100mM 3-AT. Proteins fused to the binding domain (BD) and used as bait are labelled first and those fused to the activation domain (AD) and used as prey are labelled second. Control A, B and C are explained in the ProQuest Two-Hybrid System manual and are used to show weak, intermediate and strong interactions respectively. Positive interactions are highlighted by the red dashed lines.

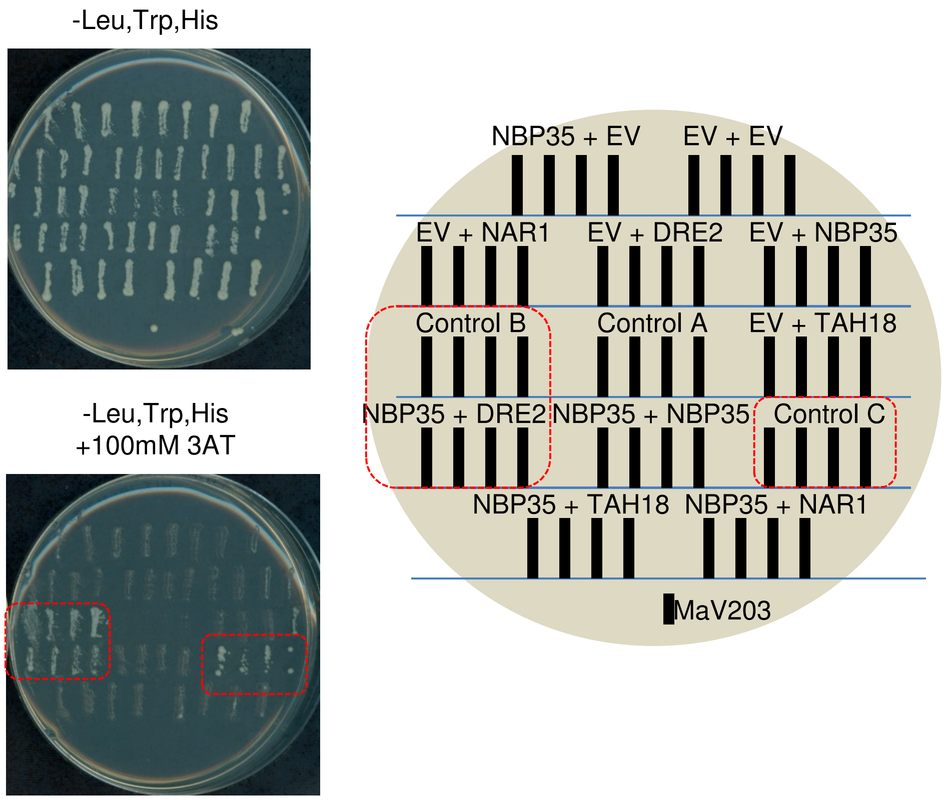

Figure S9. Model of the CIA pathway in plants.

The cysteine desulfurase (NFS1) releases sulfur from cysteine and this is exported from mitochondria via the ABC transporter (ATM3). In the cytosol, NBP35 is a scaffold protein for the assembly of FeS clusters. NBP35 forms a weak interaction with DRE2 which also interacts with TAH18 to form an electron transport chain using NAD(P)H as a substrate. The CIA complex of proteins consisting of AE7, MET18, CIA1 and NAR1 interacts with target proteins.

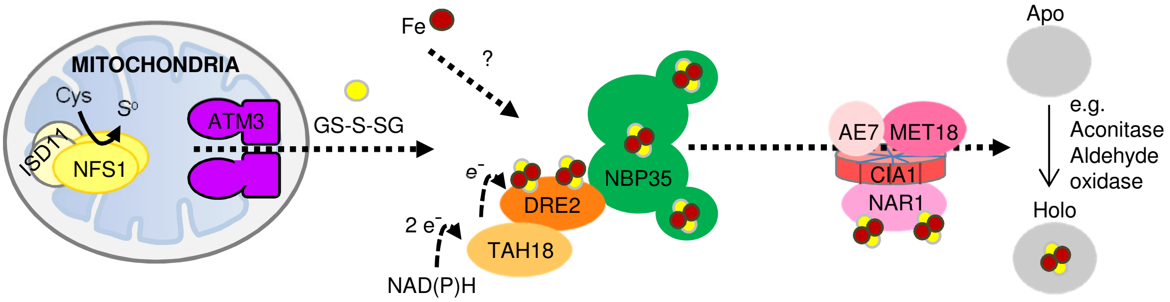

Supplement: Supplementary file 1 — Figure S1. Quantification of NPB35 protein levels in knockdown lines. Figure S2. Alignment of Saccharomyces cerevisiae and Arabidopsis thaliana NBP35. Figure S3. Root growth and transcript levels of FeS proteins in NBP35‐C14A. Figure S4. Developmental defects in plants expressing NBP35:NBP35‐C14A. Figure S5. Expression and purification of NBP35 and NBP35‐C14A. Figure S6. Spectroscopy of reconstituted NBP35 and NBP35‐C14A. Figure S7. NBP35 and DRE2 gene expression during development. Figure S8. Confirmation of yeast‐two‐hybrid interactions identified in the screen. Figure S9. Model of the CIA pathway in plants. [file TPJ-89-590-s001.pdf]
