# Supplementary material for: NBP35 interacts with DRE2 in the maturation of cytosolic iron‐sulphur proteins in Arabidopsis thaliana
Source: Plant J. 2017 Feb 3;89(3):590–600. doi: 10.1111/tpj.13409 (PMC5324674; doi:10.1111/tpj.13409)
Supplement: Supplementary file 2 — Table S1. Yeast‐two‐hybrid interactions of Arabidopsis NBP35 screened against a fragment library of seedling cDNA Table S2. List of primers. [file TPJ-89-590-s002.pptx]

## Slide 1
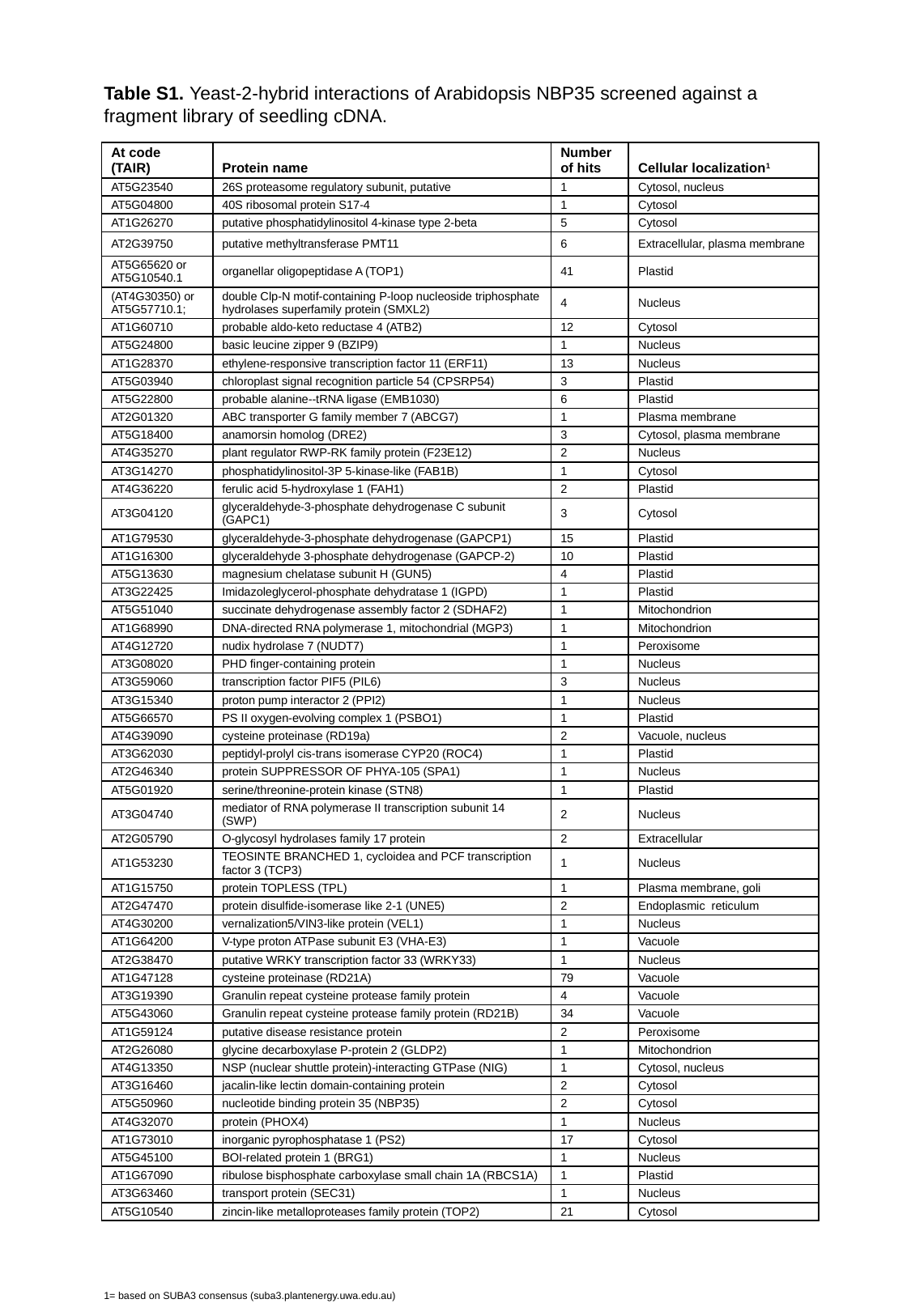

Table S1. Yeast-2-hybrid interactions of Arabidopsis NBP35 screened against a fragment library of seedling cDNA.
| At code (TAIR) | Protein name | Number of hits | Cellular localization1 |
| --- | --- | --- | --- |
| AT5G23540 | 26S proteasome regulatory subunit, putative | 1 | Cytosol, nucleus |
| AT5G04800 | 40S ribosomal protein S17-4 | 1 | Cytosol |
| AT1G26270 | putative phosphatidylinositol 4-kinase type 2-beta | 5 | Cytosol |
| AT2G39750 | putative methyltransferase PMT11 | 6 | Extracellular, plasma membrane |
| AT5G65620 or AT5G10540.1 | organellar oligopeptidase A (TOP1) | 41 | Plastid |
| (AT4G30350) or AT5G57710.1; | double Clp-N motif-containing P-loop nucleoside triphosphate hydrolases superfamily protein (SMXL2) | 4 | Nucleus |
| AT1G60710 | probable aldo-keto reductase 4 (ATB2) | 12 | Cytosol |
| AT5G24800 | basic leucine zipper 9 (BZIP9) | 1 | Nucleus |
| AT1G28370 | ethylene-responsive transcription factor 11 (ERF11) | 13 | Nucleus |
| AT5G03940 | chloroplast signal recognition particle 54 (CPSRP54) | 3 | Plastid |
| AT5G22800 | probable alanine--tRNA ligase (EMB1030) | 6 | Plastid |
| AT2G01320 | ABC transporter G family member 7 (ABCG7) | 1 | Plasma membrane |
| AT5G18400 | anamorsin homolog (DRE2) | 3 | Cytosol, plasma membrane |
| AT4G35270 | plant regulator RWP-RK family protein (F23E12) | 2 | Nucleus |
| AT3G14270 | phosphatidylinositol-3P 5-kinase-like (FAB1B) | 1 | Cytosol |
| AT4G36220 | ferulic acid 5-hydroxylase 1 (FAH1) | 2 | Plastid |
| AT3G04120 | glyceraldehyde-3-phosphate dehydrogenase C subunit (GAPC1) | 3 | Cytosol |
| AT1G79530 | glyceraldehyde-3-phosphate dehydrogenase (GAPCP1) | 15 | Plastid |
| AT1G16300 | glyceraldehyde 3-phosphate dehydrogenase (GAPCP-2) | 10 | Plastid |
| AT5G13630 | magnesium chelatase subunit H (GUN5) | 4 | Plastid |
| AT3G22425 | Imidazoleglycerol-phosphate dehydratase 1 (IGPD) | 1 | Plastid |
| AT5G51040 | succinate dehydrogenase assembly factor 2 (SDHAF2) | 1 | Mitochondrion |
| AT1G68990 | DNA-directed RNA polymerase 1, mitochondrial (MGP3) | 1 | Mitochondrion |
| AT4G12720 | nudix hydrolase 7 (NUDT7) | 1 | Peroxisome |
| AT3G08020 | PHD finger-containing protein | 1 | Nucleus |
| AT3G59060 | transcription factor PIF5 (PIL6) | 3 | Nucleus |
| AT3G15340 | proton pump interactor 2 (PPI2) | 1 | Nucleus |
| AT5G66570 | PS II oxygen-evolving complex 1 (PSBO1) | 1 | Plastid |
| AT4G39090 | cysteine proteinase (RD19a) | 2 | Vacuole, nucleus |
| AT3G62030 | peptidyl-prolyl cis-trans isomerase CYP20 (ROC4) | 1 | Plastid |
| AT2G46340 | protein SUPPRESSOR OF PHYA-105 (SPA1) | 1 | Nucleus |
| AT5G01920 | serine/threonine-protein kinase (STN8) | 1 | Plastid |
| AT3G04740 | mediator of RNA polymerase II transcription subunit 14 (SWP) | 2 | Nucleus |
| AT2G05790 | O-glycosyl hydrolases family 17 protein | 2 | Extracellular |
| AT1G53230 | TEOSINTE BRANCHED 1, cycloidea and PCF transcription factor 3 (TCP3) | 1 | Nucleus |
| AT1G15750 | protein TOPLESS (TPL) | 1 | Plasma membrane, goli |
| AT2G47470 | protein disulfide-isomerase like 2-1 (UNE5) | 2 | Endoplasmic reticulum |
| AT4G30200 | vernalization5/VIN3-like protein (VEL1) | 1 | Nucleus |
| AT1G64200 | V-type proton ATPase subunit E3 (VHA-E3) | 1 | Vacuole |
| AT2G38470 | putative WRKY transcription factor 33 (WRKY33) | 1 | Nucleus |
| AT1G47128 | cysteine proteinase (RD21A) | 79 | Vacuole |
| AT3G19390 | Granulin repeat cysteine protease family protein | 4 | Vacuole |
| AT5G43060 | Granulin repeat cysteine protease family protein (RD21B) | 34 | Vacuole |
| AT1G59124 | putative disease resistance protein | 2 | Peroxisome |
| AT2G26080 | glycine decarboxylase P-protein 2 (GLDP2) | 1 | Mitochondrion |
| AT4G13350 | NSP (nuclear shuttle protein)-interacting GTPase (NIG) | 1 | Cytosol, nucleus |
| AT3G16460 | jacalin-like lectin domain-containing protein | 2 | Cytosol |
| AT5G50960 | nucleotide binding protein 35 (NBP35) | 2 | Cytosol |
| AT4G32070 | protein (PHOX4) | 1 | Nucleus |
| AT1G73010 | inorganic pyrophosphatase 1 (PS2) | 17 | Cytosol |
| AT5G45100 | BOI-related protein 1 (BRG1) | 1 | Nucleus |
| AT1G67090 | ribulose bisphosphate carboxylase small chain 1A (RBCS1A) | 1 | Plastid |
| AT3G63460 | transport protein (SEC31) | 1 | Nucleus |
| AT5G10540 | zincin-like metalloproteases family protein (TOP2) | 21 | Cytosol |
1= based on SUBA3 consensus (suba3.plantenergy.uwa.edu.au)

## Slide 2
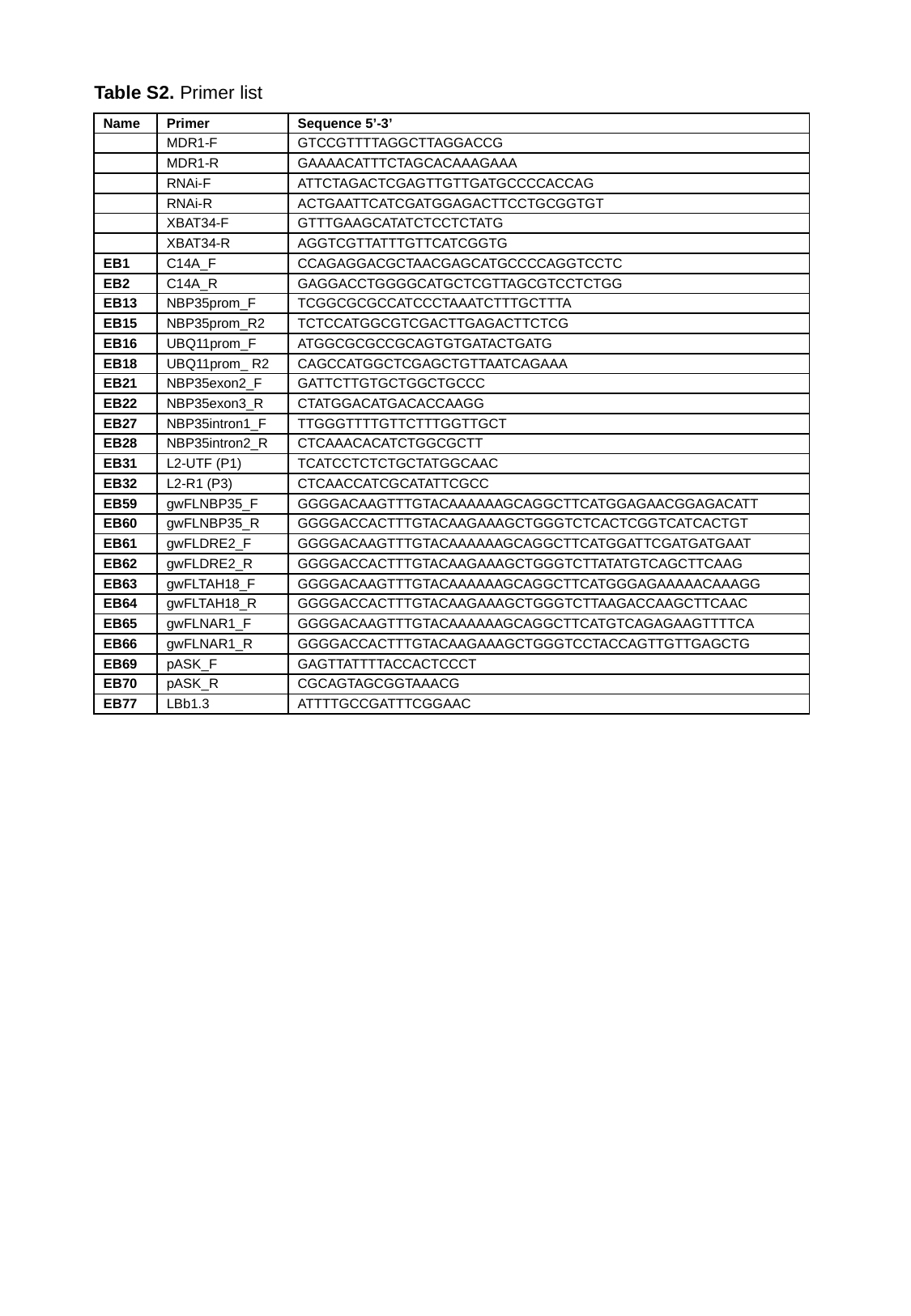

Table S2. Primer list
| Name | Primer | Sequence 5’-3’ |
| --- | --- | --- |
| | MDR1-F | GTCCGTTTTAGGCTTAGGACCG |
| | MDR1-R | GAAAACATTTCTAGCACAAAGAAA |
| | RNAi-F | ATTCTAGACTCGAGTTGTTGATGCCCCACCAG |
| | RNAi-R | ACTGAATTCATCGATGGAGACTTCCTGCGGTGT |
| | XBAT34-F | GTTTGAAGCATATCTCCTCTATG |
| | XBAT34-R | AGGTCGTTATTTGTTCATCGGTG |
| EB1 | C14A\_F | CCAGAGGACGCTAACGAGCATGCCCCAGGTCCTC |
| EB2 | C14A\_R | GAGGACCTGGGGCATGCTCGTTAGCGTCCTCTGG |
| EB13 | NBP35prom\_F | TCGGCGCGCCATCCCTAAATCTTTGCTTTA |
| EB15 | NBP35prom\_R2 | TCTCCATGGCGTCGACTTGAGACTTCTCG |
| EB16 | UBQ11prom\_F | ATGGCGCGCCGCAGTGTGATACTGATG |
| EB18 | UBQ11prom\_ R2 | CAGCCATGGCTCGAGCTGTTAATCAGAAA |
| EB21 | NBP35exon2\_F | GATTCTTGTGCTGGCTGCCC |
| EB22 | NBP35exon3\_R | CTATGGACATGACACCAAGG |
| EB27 | NBP35intron1\_F | TTGGGTTTTGTTCTTTGGTTGCT |
| EB28 | NBP35intron2\_R | CTCAAACACATCTGGCGCTT |
| EB31 | L2-UTF (P1) | TCATCCTCTCTGCTATGGCAAC |
| EB32 | L2-R1 (P3) | CTCAACCATCGCATATTCGCC |
| EB59 | gwFLNBP35\_F | GGGGACAAGTTTGTACAAAAAAGCAGGCTTCATGGAGAACGGAGACATT |
| EB60 | gwFLNBP35\_R | GGGGACCACTTTGTACAAGAAAGCTGGGTCTCACTCGGTCATCACTGT |
| EB61 | gwFLDRE2\_F | GGGGACAAGTTTGTACAAAAAAGCAGGCTTCATGGATTCGATGATGAAT |
| EB62 | gwFLDRE2\_R | GGGGACCACTTTGTACAAGAAAGCTGGGTCTTATATGTCAGCTTCAAG |
| EB63 | gwFLTAH18\_F | GGGGACAAGTTTGTACAAAAAAGCAGGCTTCATGGGAGAAAAACAAAGG |
| EB64 | gwFLTAH18\_R | GGGGACCACTTTGTACAAGAAAGCTGGGTCTTAAGACCAAGCTTCAAC |
| EB65 | gwFLNAR1\_F | GGGGACAAGTTTGTACAAAAAAGCAGGCTTCATGTCAGAGAAGTTTTCA |
| EB66 | gwFLNAR1\_R | GGGGACCACTTTGTACAAGAAAGCTGGGTCCTACCAGTTGTTGAGCTG |
| EB69 | pASK\_F | GAGTTATTTTACCACTCCCT |
| EB70 | pASK\_R | CGCAGTAGCGGTAAACG |
| EB77 | LBb1.3 | ATTTTGCCGATTTCGGAAC |
